# *Monascus* red pigment influence on hydroxyapatite nanoparticles-mediated renal toxicity in rats

**DOI:** 10.1038/s41598-024-84959-z

**Published:** 2025-01-21

**Authors:** Nasser N. Alqurashy, Mokhtar I. Yousef, Ahmed A. Hussein, Maher A. Kamel, Abeer El Wakil

**Affiliations:** 1 Department of Biotechnology, Institute of Graduate Studies and Research, Alexandria University, Alexandria, Egypt; 2 Department of Environmental Studies, Institute of Graduate Studies and Research, Alexandria University, Alexandria, Egypt; 3 Department of Biochemistry, Medical Research Institute, Alexandria University, Alexandria, Egypt; 4 Department of Biological and Geological Sciences, Faculty of Education, Alexandria University, Alexandria, 21526 Egypt

**Keywords:** Natural products, Biopigment, Oxidative stress, Kidney, Biotechnology, Nephrology, Nanoscience and technology

## Abstract

Hydroxyapatite nanoparticles (HANPs) have been applied in several biomedical fields. However, its interaction with biological systems is less exploited. This study aimed to characterize HANPs, examine their influence on kidneys, and explore the potential protective effects of naturally extracted red pigment (RP) from *Monascus purpureus* against HANPs-induced renal toxicity. To this aim, forty eight adult male rats were randomly divided into 8 equal groups: a control group receiving 4% dimethyl sulfoxide (the solvent for HANPs), three groups receiving extracted RP at different doses of 10, 20, and 40 mg/kg, a group receiving HANPs at a dose of 88.3 mg/kg, and three more groups receiving a double treatment of HANPs associated with RP. The respective treatment was given daily by oral gavage to animals for 50 days which is the duration of the whole experiment. The renal toxicity caused by HANPs was manifested by aberrations in kidney function parameters, intensification of oxidative stress markers, and a decrease in the activity of antioxidant enzymes. Moreover, an increase in inflammatory (TNF-α and TGF-β) and apoptotic (caspace-3) markers, an elevation in gene-based kidney injuries markers (Kim-1 and lipocalin-2), and pathological tissue changes were determined. Meanwhile, co-treatment with different doses of biopigment and HANPs have reduced oxidative stress via their potent antioxidant effect. This was confirmed by pronounced improvement in the measured parameters along with the histological structural enhancement in a dose dependent manner compared to controls. To sum up, RP from *M. purpureus* has potential protective benefits in mitigating the adverse effects of HANPs.

## Introduction

Despite nanotechnology’s popularity is increasing in medicine, its applications have been limited due to its possible toxicity and long-term secondary detrimental consequences^1^. The physio-chemical unique characteristics of nanoparticles (NPs) such as small sized particles, large surface area-to-volume ratio, and the malleable chemical structure/composition support their application in nanomedicine. However, in the meantime, they played a role in NPs increased toxicological adverse effects^2–5^. Hydroxyapatite nanoparticles (HANPs) are among the most important members of ultrafine materials which have called attention due to their biocompatibility, bioactivity, and osteoconductivity as they often serve as bone substitute in intra-osseous implantation or as implant coating materials^6,7^. With the increased applications of HANPs, the concerns about their potential human toxicity and their environmental impact stuck out. HANPs-mediated toxicity may be due to the oxidative stress mechanism occurring via both free radicals and reactive oxidative species (ROS).

Kidneys play various vital functions to maintain normal homeostasis within the body. The major renal contributions include excretion of waste products of metabolism, regulation of body water and salt, maintenance of extracellular fluid volume, maintenance of acid–base balance, and elimination of foreign substances such as drugs and chemicals and their breakdown products^8^. High blood supply and ability to concentrate toxins, makes the kidneys particularly susceptible to xenobiotics, including NPs, which reach the bloodstream. Additionally, kidneys are recognized as one of the most vulnerable target organ to the toxic effects of drugs and environmental chemicals^9^. Any alterations in the capacity of renal functions may cause detrimental effects in the whole body^10^.

Although the kidneys are the primary organ of xenobiotic elimination in the body, yet a little attention has been paid to them in terms of nanotoxicological research uptill now. Understanding renal aberrations with respect to functional anatomy, pathophysiology, biomarkers of nephrotoxicity, and alternative models that allow human translation of preclinical data is very critical for pharmaceutical drug development, chemical hazard risk assessment, and consumer health industries^8^.

Usage of synthetic dyes in the field of food production, pharmaceutical, and textile industries posed critical environmental and health issues^11^. Consequently, biocolorants offer natural and good alternatives to replace hazardous artificial coloring agents. Plants and microorganisms are the major source for biopigments^12^. Importantly, microorganisms are superior over plants as they are not only sustainable alternatives but also renewable sources accompanied with immense production. They are rapidly growing organisms, cost-effective, easily manipulated genetically, simplicity in handling, and no need for big land areas for their growth like plants. Biopigments, either red (rubropanctamine and monascorubramine) or orange (monascorubrin and rubropunctatin), are produced by nine species of the fungal genus *Monascus*^13^. *Monascus purpureus* red pigments (RP) are used as potential therapeutic agents owing to their varied biological activities such as anti-inflammatory, anti-tumor, antioxidant and regulating action of high cholesterol levels. They are also characterized by their high stability against high temperatures and pH alteration^14^.

Herein, we investigated the cytotoxic and genotoxic potential of HANPs on the kidneys of 40 Wistar male rats. Our aim was to assess the protective potential of different doses of RP against HANPs-mediated nephrotoxicity.

## Materials and methods

### Chemicals and materials

HANPs (white nano powder, size = 100 ± 30 nm, length = 20 ± 5 nm) were obtained from Nanotech Egypt Co. (Giza, Egypt). All chemicals were of high quality and analytical grade. ELISA kits were procured from Abcam (24 rue Louis Blanc, Paris, France).

### Characterization of HANPs

Purchased HANPs were characterized by different tools including (i) high resolution-transmission electron microscopy (HR-TEM), which is an imaging mode of TEM that allows the imaging of the structure of a sample at an atomic scale. JEM-2100Plus (JEOL Ltd, Japan), operated at an accelerating voltage of 80 kV depending on the type and thickness of the specimen^15,16^, is a versatile TEM for both large-scale 2D screening as well as tomography. It runs serial EM and it is thus capable of automated multiposition acquisitions. (ii) Fourier Transform Infrared (FTIR) spectrums were measured using a Shimadzu FTIR-8400 Spectrometer (Shimadzu, Japan), recorded in the wavelength range 400–4000 cm^− 1^. Dried samples of about 100 mg were mixed with 100 mg of spectral grade potassium bromide (KBr) and pressed into discs under hydraulic pressure^17^. (iii) Finally, X-ray diffraction (XRD) patterns of the samples were obtained using Schimadzu 7000 Diffractometer (Shimadzu, Japan) operating with CuKα radiation (λ = 0.15406 nm) generated at 30 kV and 30 mA with scan rate of 4º min^− 1^ for 20 values between 4 and 80 degrees.

### Microorganisms, fermentation, and extraction of the natural red pigment

*Monascus purpureus* strain ATCC16436, as a producer for RP used in this study, was obtained from Microscobiolgical Resources Center (MIRCEN, Cairo, Egypt) for Egypt microbial culture collection (EMCC). RP of *Monascus purpureus* was prepared according to Zhou et al.^18^ in the Microbiology Laboratory of the Biotechnology Department at the Institute of Graduate Studies and Research, Alexandria University, Egypt. Rice grains were purchased from local markets in Alexandria city (Alexandria, Egypt). The modified minimal medium (0.5 g NH_4_SO_4_, NH_4_NO_3_, KNO_3_, peptone, 0.2 g KH_2_PO_4_, K_2_HPO_4_, 0.2 g MgSO_4_, 0.001 mM ZnSO_4_, 0.002 mM MnSO_4_) were used as a core medium for *Monascus* pigments biosynthesis to which 3 g of rice grains were added per each 100 mL media^19^. The final pH of the medium was adjusted to 4.5. Potato dextrose agar plates were inoculated with the fungal spores. Then, the inoculated plates were incubated at 30 °C for 7 days in a static incubator. A suspension of fungal spores prepared using sterile water was diluted and adjusted to 2 × 10^4^ by hemocytometer. *Monascus* pigments were extracted according to a slight modification of the previously reported procedure^20^. At the end of the incubation period, 20 mL of 96% ethanol was added to the fermented preparation. Then, the mixture was incubated for 2 h with an agitation speed of 180 rpm. After that, the mixture was filtered through Whatman paper #1.0. The filtrate was further centrifuged at 10,000 rpm for 10 min. The supernatant was taken and kept at 4°C until being processed further.

### Characterization of the natural red pigment

The extracted red pigment from *M. purpureus* was characterized by (i) Ultraviolet-Visible (UV-Vis) spectrum obtained from the chromatogram after background subtraction. It was detected on a spectrophotometer in the range 300–700 nm, (ii) Liquid chromatography-mass spectrometer (LC-MS) analysis was carried out using an Ace C18-AR column (3 μm 100 Å, 50 × 4.6 mm). Moreover, 0.1% formic acid in water and 0.1% formic acid in acetonitrile were used at a flow rate 0.5 mL/min as mobile phases A and B, respectively. The gradient under the chromatographic conditions was programmed as follows: 0–1 min (95% A); 1–3 min (80% A); 3–7 min (0% A); 7–9 min (0% A); 9–9.1 min (95% A); and 9.1–14 min (95% A). The injection volume was 3 µl for each of the sample solutions. The column temperature was maintained at 30 °C. The MS conditions were applied using the Q Exactive™ Orbitrap instrument (Thermo-Fischer Scientific, USA), and (iii) FTIR analysis was carried out to determine the purity of the extracted pigment using FTIR spectrophotometer (PerkinElmer, USA). All spectra were acquired with 32 scans at 4 cm^− 1^ resolution within a wavenumber range of 350–4000 cm^− 1^ at room temperature.

### Animal care and experimental set up

Forty eight adult male Wistar rats weighing from 180 to 200 g were obtained from the Faculty of Medicine, Alexandria University, Egypt. They were housed under standard laboratory conditions (25 ± 5 °C, 50–60% humidity) and maintained on a 12 h light:12 h dark photoperiod cycle with free access to basal diet and tap water ad libitum. After acclimatization for 2 weeks, the animals were randomly divided into 8 equal groups, each receiving a particular treatment. The applied dose of HANPs was 88.3 mg/kg body weight according to Nair and Jacob^21^, while the approved dose of the extracted red pigment of *Monascus purpureus* was 20 mg/kg body weight according to Zhou et al.^18^ which we considered as an intermediate dose in the present study. In the meantime, we investigated the administration of a low dose of RP from *Monascus purpureus* corresponding to half of the approved dose, i.e. 10 mg/kg body weight, as well as a high dose of RP equalling twice of the approved dose, i.e. 40 mg/kg body weight. The treatment groups were distributed as follows: (1) Group 1 rats were the controls and received 4% dimethyl sulfoxide (DMSO) which is the solvent for HANPs; (2) Group 2 rats received a dose of 10 mg RP/kg; (3) Group 3 rats received a dose of 20 mg RP/kg; (4) Group 4 rats received a dose of 40 mg RP/kg; (5) Group 5 animals were given HANPs at a dose of 88.3 mg/kg; (6) Group 6 animals were given 10 mg RP/kg co-supplemented with HANPs (88.3 mg/kg); (7) Group 7 rats received a combinatory treatment of 20 mg RP/kg and HANPs (88.3 mg/kg); and (8) Group 8 rats received a double treatment with 40 mg RP/kg and HANPs (88.3 mg/kg). The respective treatment were given daily by oral gavage to animals and continued for 50 days which is the duration of the whole experiment.

All experimental procedures were carried out in accordance with ARRIVE guidelines and the Guide for the Care and Use of Laboratory animals (International Council for Laboratory Animal Science, ICLAS) under the approval number AU14-211017-2-10 provided by the institutional animal care and use committee (IACUC) at Alexandria University, Egypt.

### Blood and organ collection

At the end of the experiment, rats were fasted overnight and sacrificed under anesthesia by inhalation of 3% isoflurane, and every effort was made to minimize suffering. Blood was collected by cardiac puncture in test tubes containing heparin. Plasma was obtained from whole blood by centrifugation at 860 x g for 20 min. Aliquots of plasma were preserved at -20 °C until analysis. Both kidneys were removed, released from the adhering fat and connective tissues, washed by chilled saline solution (0.9%), and dried on tissue paper. The left kidney was immediately kept in 10% formalin for histological studies. Meanwhile, the right kidney was minced and homogenized (10%, w/v), separately, in ice-cold phosphate buffer (0.25 M, pH 7.4) in a Potter–Elvehjem type homogenizer. Homogenates were centrifuged at 10,000 x g for 20 min at 4 °C to pellet cell debris, and the supernatant was collected and stored at − 80 °C for further analyses.

### Determination of kidney function parameters

Plasma urea, creatinine and uric acid are the most widely accepted parameters to evaluate kidney dysfunction status. They were determined and quantified spectrophotometrically using commercially available kits from Biosystems S.A., Barcelona, Spain. Measurements of these parameters were performed strictly according to the manufacturer’s instructions.

### Assessment of renal antioxidants and oxidative stress biomarkers

Malondialdehyde (MDA), a lipid peroxidation marker, was determined following the method of Draper and Hadley^22^, while nitrite and nitrate (NOx) levels were measured by Griess reaction^23^. The enzymatic method described by Griffith^24^ was used to evaluate the oxidative stress status in biological tissues by quantifying the total glutathione (GSH) and glutathione disulfide (GSSG) contents. Superoxide dismutase (SOD) and total glutathione peroxidase (GPx) activities were determined by pyrogallol method of Marklund and Marklund^25^ and Flohé and Günzler^26^ method, respectively. Measurements of these parameters were performed in renal tissue homogenates.

### Measurement of oxidative DNA damage index

8-Hydroxy-2-deoxyguanosine (8-OHdG), a pivotal marker measuring the effect of endogenous oxidative damage to DNA, was assayed using Rat 8-OHdG ELISA Kit (Cat # ab285302) according to the product’s manual (Abcam, USA).

### Evaluation of renal inflammatory and apoptotic markers in kidney tissues by enzyme-linked immunosorbent assay (ELISA)

Quantitative measurements, in the supernatant of rat kidney homogenates, of tumor necrosis factor-alpha (TNF-α), and transforming growth factor-beta (TGF-β) were performed according to the instructional manuals of their respective ELISA kits (Biospes Co., China). The caspase-3 enzymatic activity was assayed using Caspase-3 Assay Kit from Elabscience (USA) in compliance with the manufacturer’s protocol.

### Gene expression analysis of kidney injury molecule-1 (Kim-1) and lipocalin-2 using quantitative real time-polymerase chain reaction (qRT-PCR)

Kidney total RNA was isolated using RNeasy mini kit (Qiagen, Hilden, Germany) and reverse transcribed into cDNA using miScript II reverse transcriptase (Qiagen, Hilden, Germany). Real-time PCR using specific primer sets for Kim-1 (Accession Nº NM_173149.2; sense: TGG CAC TGT GAC ATC CTC AGA, antisense: GCA ACG GAC ATG CCA ACA TA) and lipocalin-2 (Accession Nº NM_130741.1; sense: GGA ATA TTC ACA GCT ACC CTC, antisense: TTG TTA TCC TTG AGG CCC AG] was performed using SYBR green (Qiagen, Hilden, Germany) and 18s rRNA (Accession Nº NR_046237.2; sense primer: GTA ACC CGT TGA ACC CCA TT; antisense primer: CAA GCT TAT GAC CCG CAC TT) as a reference gene in a LightCycler machine (Roche, Basel, Switzerland).

### Histopathology examination

Tissue sections of fixed kidneys (10% formalin ) at 4–6 μm thickness were mounted on poly-L-lysine-coated slides, deparaffinized in xylene, and rehydrated through ethanol series. They were then stained with hematoxylin and counterstained with eosin for histological examination. Photographs were taken on an Olympus XC30 microscope (Germany) with a digital camera (Olympus UC30 camera). Representative pictures were taken at 100x and 400x magnification from each group.

### Statistical analysis

Animals within the different groups were compared for differences by ANOVA multiple comparisons. Afterwards, Tukey kramer as a post-hoc test was performed. All data were presented as means ± SD. Statistical significance was judged at the 5% level using F-test. All procedures were performed using IBM SPSS software.

## Results

### Tools for HANPs characterization

**Fig. 1:**
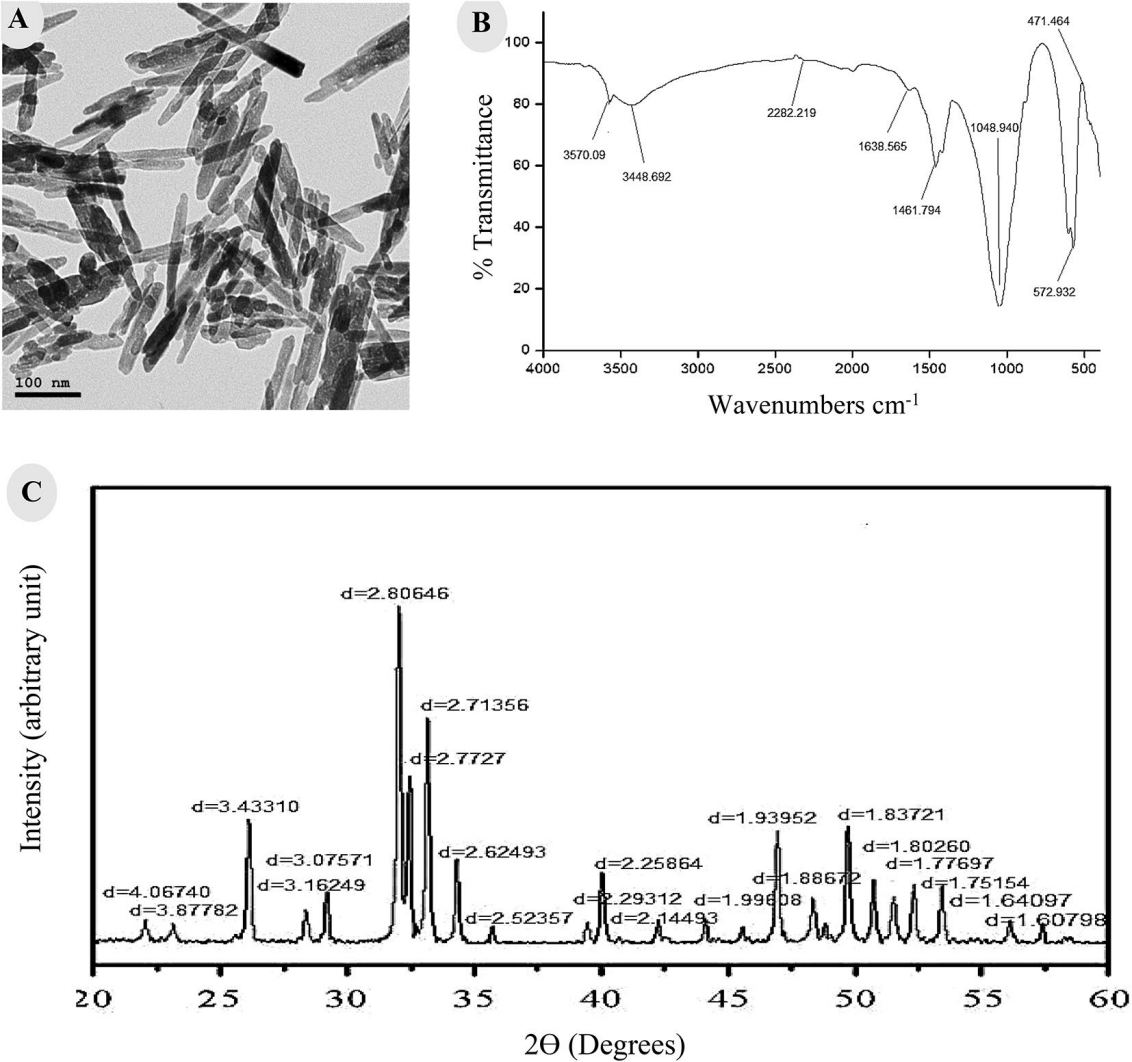
HANPs characterization showing (**A**) A TEM micrograph of the rod-like crystal-shaped appearance of nanoparticles, (**B**) FTIR spectrum revealing the presence of phosphate (PO_4_^3−^), carbonate (CO_3_^2−^) and hydroxyl (^−^OH) groups with various peaks, and (**C**) XRD pattern of crystallites.

The structure and morphology of the HANPs samples were analyzed by TEM (Fig. 1A). Images clearly show that they are rod-like shaped in appearance with an average size of 100 ± 30 nm. Functional groups associated with HANPs were identified by FTIR spectroscopy (Fig. 1B). FTIR spectrum revealed the presence of phosphate (PO_4_^3−^), carbonate (CO_3_^2−^) and hydroxyl (^−^OH) groups with various peaks. The sharp narrow band at 3570.09 and wide band at 3448.69 cm^− 1^ are associated with hydroxyl group. These two peaks prove the presence of HA phase. The intensity band at about 1461.79 and 1638.565 cm^− 1^ in the spectrum of HANPs are attributed to components of a trace amount of CO_3_^2−^. The highest intensity in PO_4_^3−^ is detected at 471.464, 672.932, and 1048.94 cm^− 1^ by the vibration bonding and P-O stretching vibration from phosphate groups.

In terms of the XRD analysis of the skeleton structure in semi-crystalline and crystalline polymers, the pattern was interpreted as crystal. No impurities have been observed confirming that the crystal is the chief inorganic phase of the sample (Fig. 1C).

### Extraction and characterization of natural RP from *Monascus purpureus*

RP was maximally extracted by ethanol from the fermentation broth and isolated following the abovementioned protocol. The supernatant was concentrated by evaporation at 40 °C in a rotary evaporator. The pigment was then obtained by freeze-drying.

**Fig. 2:**
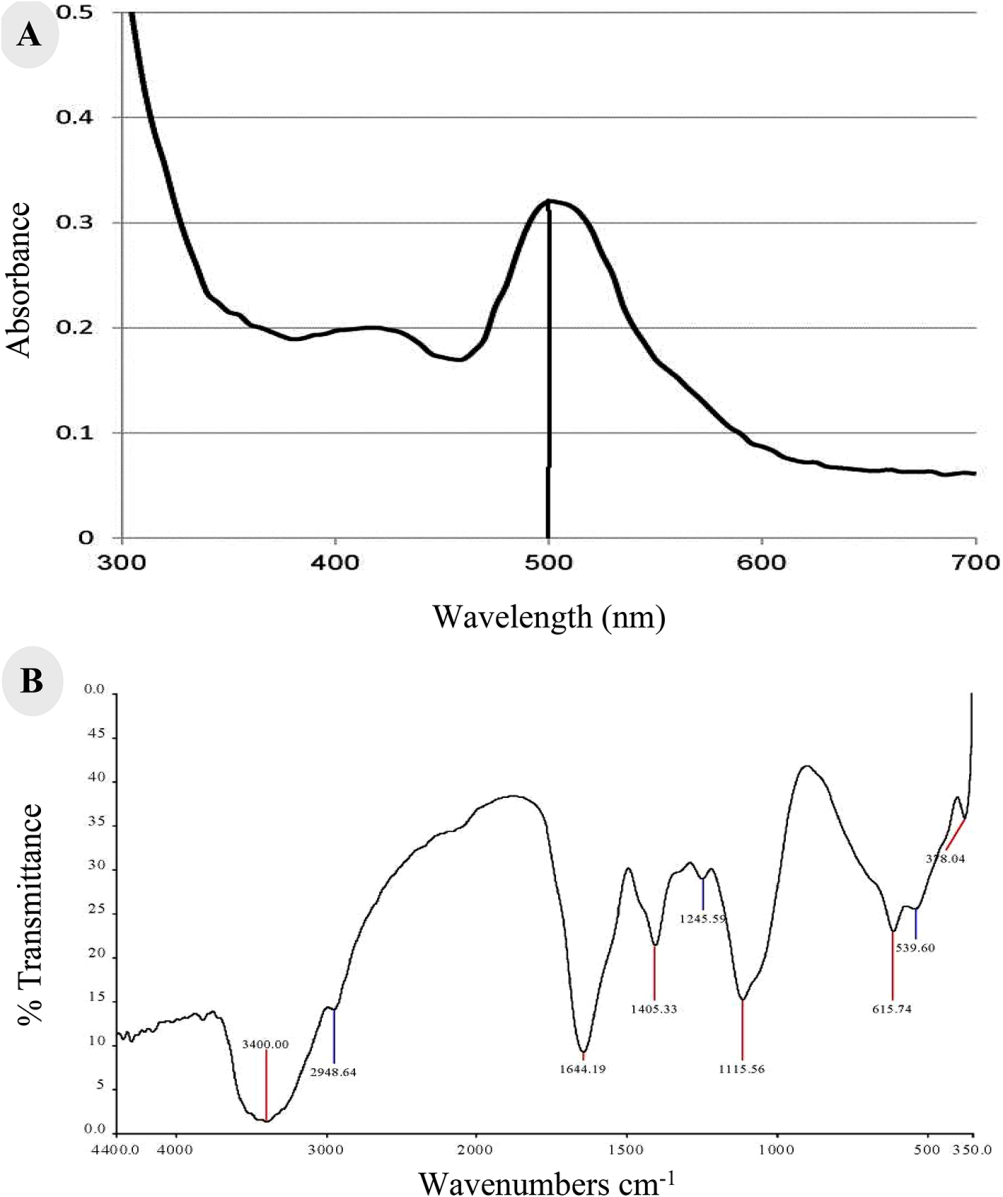
Characterization of RP from *Monascus purpureus* showing (**A**) UV–Vis spectrum, and (**B**) FTIR spectrum.

The extracted RP was characterized by UV–Vis spectrophotometry in the range 300–700 nm. Ethanol was used as control. Spectral analysis detected that X_max_ at 500 nm corresponds to RP (Fig. 2A). A thin layer chromatogram for the natural RP is observed as a single red spot under direct visualization, with a retardation factor Rƒ value of 0.69. Moreover, the chemical composition of the RP was validated by LC- Q Exactive™ Orbitrap MS system (Thermo-Fischer Scientific, USA) at 383.4 (M + H) Da which is consistent with the expected value for the molecular mass of RP from *Monascus purpureus*.

FTIR absorption of the RP was characterized by strong and broad band at 3400 cm^− 1^ indicating the presence of N-H stretching of secondary amide. The band at 2948.64 cm^− 1^ indicates C–H stretching of CH_3_ group, while the presence of a sharp band at 1644.19 cm^− 1^ confirms the presence of carbonyl group and C=C stretching at 1405,33 cm^− 1^ (Fig. 2B).

### Evaluation of kidney function parameters under different treatment protocols

**Fig. 3:**
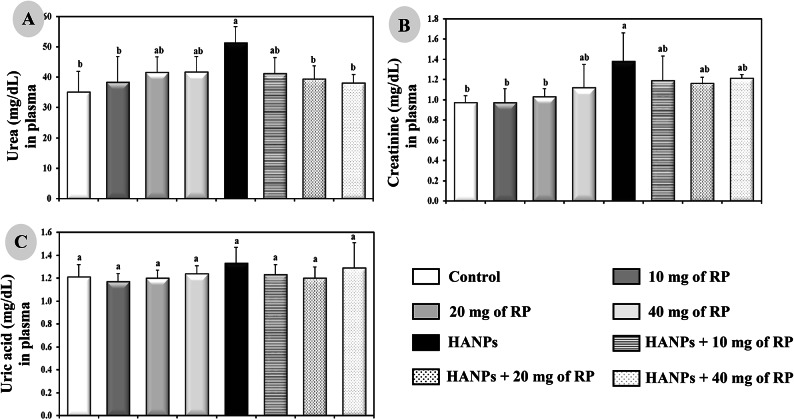
Determination of kidney function parameters illustrated by the levels of (**A**) Urea, (**B**) Creatinine, and (**C**) Uric acid in the plasma of male rats treated with different doses of RP, HANPs, and their combination for 50 days. Data are represented as mean ± SD. The same letter denotes no significant difference among experimental groups tested within each parameter separately, (*p* ≤ 0.05; *n* = 6; LSD, ANOVA test).

Rats receiving different doses of RP of *Monascus purpureus* showed no significant changes in the plasma urea, creatinine, and uric acid levels compared to the controls (Fig. 3A–C). On the other side, HANPs-treated animals showed significant increase (*p* ≤ 0.05) in plasma levels of only urea (51.2 ± 5.43 mg/dL) and creatinine (1.38 ± 0.28 mg/dL) but not in uric acid (1.33 ± 0.14 mg/dL) compared to the control rats (35.1 ± 6.84, 0.97 ± 0.07, and 1.21 ± 0.11 mg/dL, respectively). The combinatory treatment of HANPs with different doses of RP resulted in significant decrease (*p* ≤ 0.05) and completely normalized the plasma levels of urea (41.2 ± 5.25 mg/dL for 10 mg RP/kg; 39.3 ± 4.44 mg/dL for 20 mg RP/kg; and 38 ± 2.82 mg/dL for 40 mg RP/kg) and creatinine (1.19 ± 0.24 mg/dL for 10 mg RP/kg; 1.16 ± 0.06 mg/dL for 20 mg RP/kg; and 1.21 ± 0.04 mg/dL for 40 mg RP/kg) compared to the animals treated with HANPs alone (Fig. 3A–C).

### Assessment of renal oxidative stress biomarkers in the different experimental animal groups

**Fig. 4:**
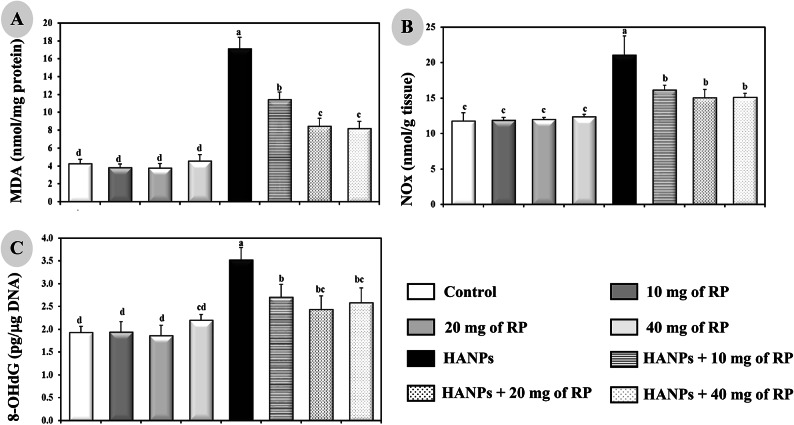
Determination of renal oxidative stress markers levels of (**A**) MDA, (**B**) NOx, and (**C**) 8-OHdG in male rats treated with different doses of RP, HANPs, and their combination for 50 days. Data are represented as mean ± SD. The same letter denotes no significant difference among experimental groups tested within each parameter separately, (*p* ≤ 0.05; *n* = 6; LSD, ANOVA test).

Administration of different doses of RP to rats showed no significant effects on the oxidative markers, mainly MDA, NOx, and 8-OHdG, compared to the controls (Fig. 4A–C). Meanwhile, HANPs-treated rats manifested notable significant increase (*p* ≤ 0.05) in the levels of renal MDA (17.12 ± 1.28 nmol/g tissue), NOx (21.05 ± 2.70 nmol/mg protein), and 8-OHdG (3.52 ± 0.27 pg/µg DNA) compared to controls (4.25 ± 0.50 nmol/g tissue, 11.75 ± 1.21 nmol/mg protein, 1.93 ± 0.13 pg/µg DNA, respectively). The double treatment with HANPs and different doses of RP significantly decreased (*p* ≤ 0.05) the renal levels of MDA (11.40 + 0.89 nmol/g tissue for 10 mg RP/kg, 8.46 + 0.89 nmol/g tissue for 20 mg RP/kg, and 8.16 + 0.84 nmol/g tissue for 40 mg RP/kg), NOx (16.11 + 0.72 nmol/mg protein for 10 mg RP/kg, 15.04 + 1.20 nmol/mg protein for 20 mg RP/kg, and 15.09 + 0.60 nmol/mg protein for 40 mg RP/kg), and 8-OHdG (2.70 + 0.28 pg/µg DNA for 10 mg RP/kg, 2.43 + 0.30 pg/µg DNA for 20 mg RP/kg, and 2.58 + 0.33 pg/µg DNA for 40 mg RP/kg) compared to the animals treated with HANPs alone (17.12 ± 1.28 nmol/g tissue, 21.05 ± 2.70 nmol/mg protein, 3.52 ± 0.27 pg/mg DNA, respectively).

### Determination of renal antioxidant enzymes activities in rats receiving different treatments

**Fig. 5:**
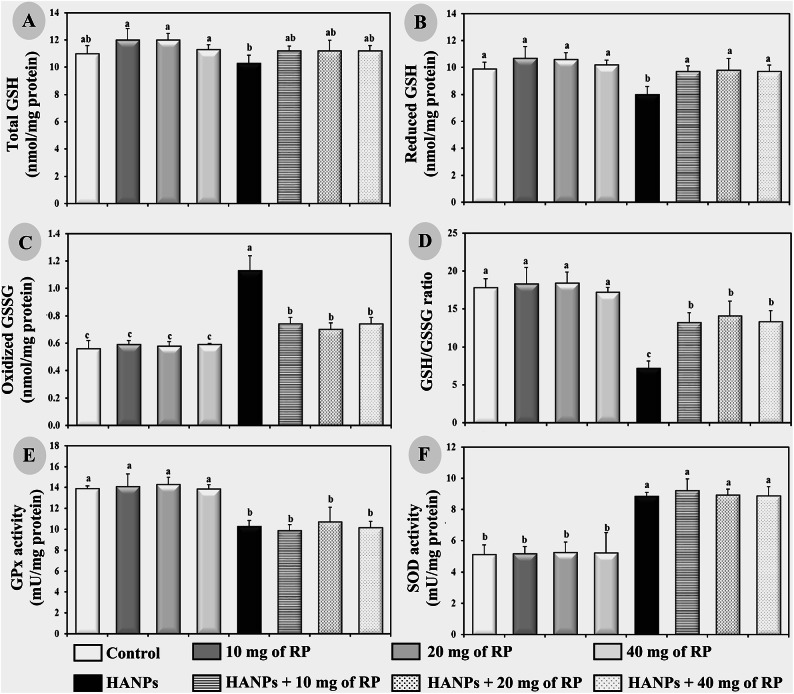
Determination of renal antioxidant enzymes activities showing (**A**) Total GSH, (**B**) Oxidized GSSG, (**C**) Reduced GSH, (**D**) GSH/GSSG ratio, (**E**) GPx, and (**F**) SOD in male rats treated with different doses of RP, HANPs, and their combination for 50 days. Data are represented as mean ± SD. The same letter denotes no significant difference among experimental groups tested within each parameter separately, (*p* ≤ 0.05; *n* = 6; LSD, ANOVA test).

Compared to the negative influence of different doses of RP on renal glutathione parameters and antioxidant enzymes activities (Fig. 5A–F), our results showed a significant decrease (*p* ≤ 0.05) in the kidney contents of reduced GSH, GSH/GSSG ratio, and the activity of GPx, whereas a significant increase (*p* ≤ 0.05) in the content of oxidised GSSG and the activity of SOD were observed in HANPs-treates rats compared to the control ones. The HANPs treatment associated with different doses of RP revealed a significant increase (*p* ≤ 0.05) in the renal contents of reduced GSH and GSH/GSSG ratio, while a significant decrease (*p* ≤ 0.05) in the GSSG content was observed compared to animals treated with HANPs alone (Fig. 5B–D). Remarkably, the HANPs double treatment with different doses of RP has no effect on the activities of SOD and GPx enzymes compared to animals treated with HANPs alone (Fig. 5E,F).

### Effects of RP on HANPs-mediated inflammatory and apoptotic markers in kidney tissues of rats under different experimental conditions

**Fig. 6:**
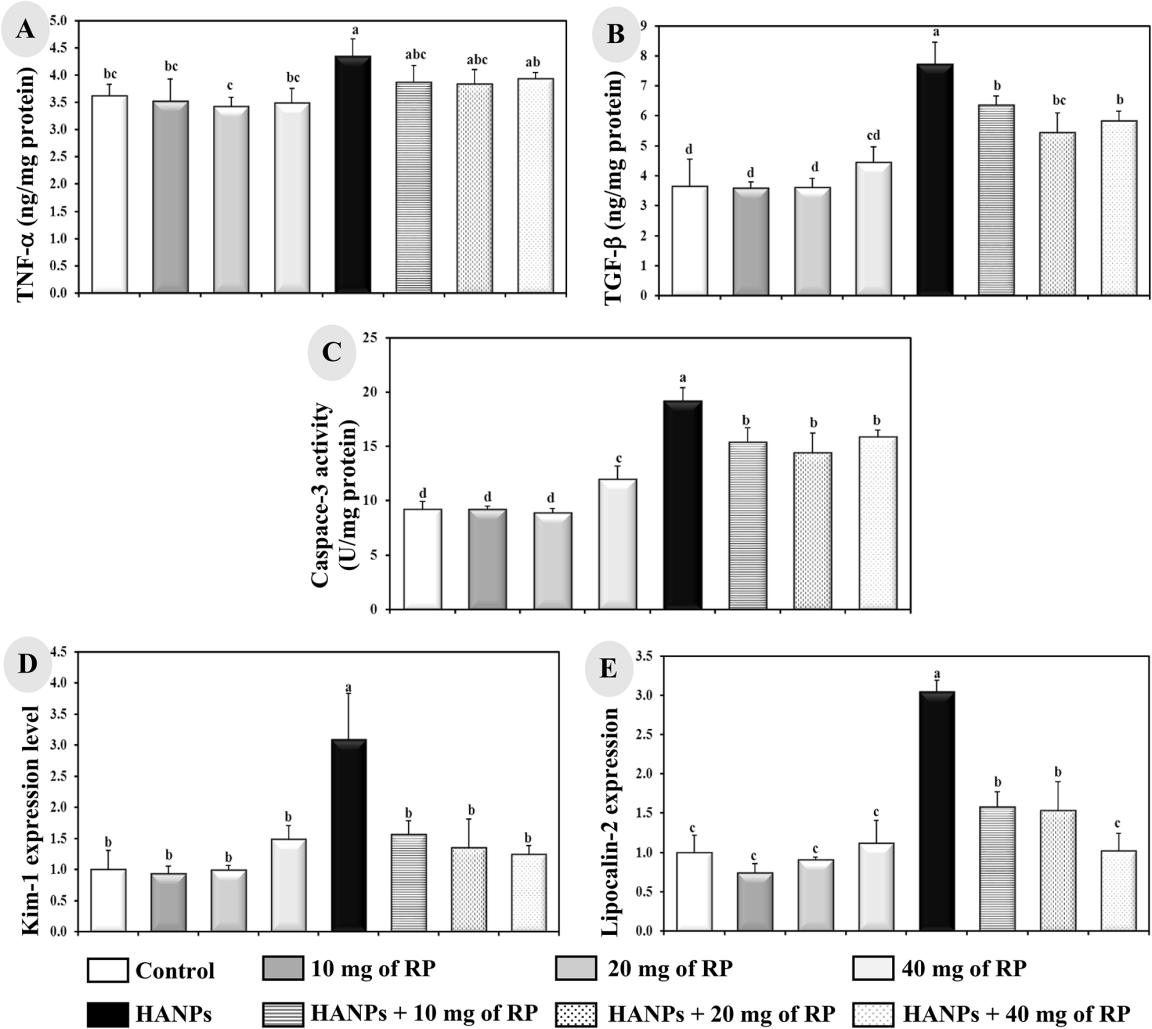
Determination of inflammatory markers including (**A**) TNF-a and (**B**) TGF-b, apoptotic marker showing (**C**) Caspace-3 as well as the expression of kidney injury genes including (**D**) Kim-1 and (**E**) lipocalin-2 in renal tissues of male rats treated with different doses of RP, HANPs, and their combination for 50 days. Data are represented as mean ± SD. The same letter denotes no significant difference among experimental groups tested within each parameter separately, (*p* ≤ 0.05; *n* = 6; LSD, ANOVA test).

Treatment with different doses of RP has no influence on the concentrations of inflammatory markers including TNF-α and TGF-β as well as the activity of the apoptotic marker, caspase-3, compared to the controls (Fig. 6A–C). The HANPs-treated rats manifested a significant increase (*p* ≤ 0.05) in the renal levels of TNF-α (4.34 ± 0.32 ng/mg protein) and TGF-β (7.72 ± 0.73 ng/mg protein) and caspase-3 activity (19.2 ± 1.18 U/mg protein) compared to control animals (3.62 ± 0.21 ng/mg protein, 3.64 ± 0.92 ng/mg protein, and 9.2 ± 0.76 U/mg protein, respectively). Combinatory treatment with HANPs and different doses of RP decreased significantly (*p* ≤ 0.05) the renal levels of TNF-α, TGF-β, and caspase-3 activity compared to the rats treated with HANPs alone. Importantly, the variation between the effects of the different doses of RP associated with HANPs treament on the inflammatory and apoptotic markers in kidney tissues of rats is neglectable.

### Expression analysis of kidney injury genes, Kim-1 and lipocalin-2, in rats following different treatments

Administration of different doses of RP has no impact on the renal gene expression levels of Kim-1 and lipocalin-2 compared to controls (Fig. 6D,E). However, HANPs administration exhibited a 3-fold increase (*p* ≤ 0.05) in the expression levels of both kim1 and lipocalin-2 compared to control animals. On the other hand, the combinatory treatment with HANPs and different doses of RP normalized (*p* ≤ 0.05) completely the expression levels of Kim-1 and lipocalin-2 compared compared to HANPs-treated rats.

### Effects of variable doses of RP, alone or in combination with HANPs, on renal histopathology

**Fig. 7:**
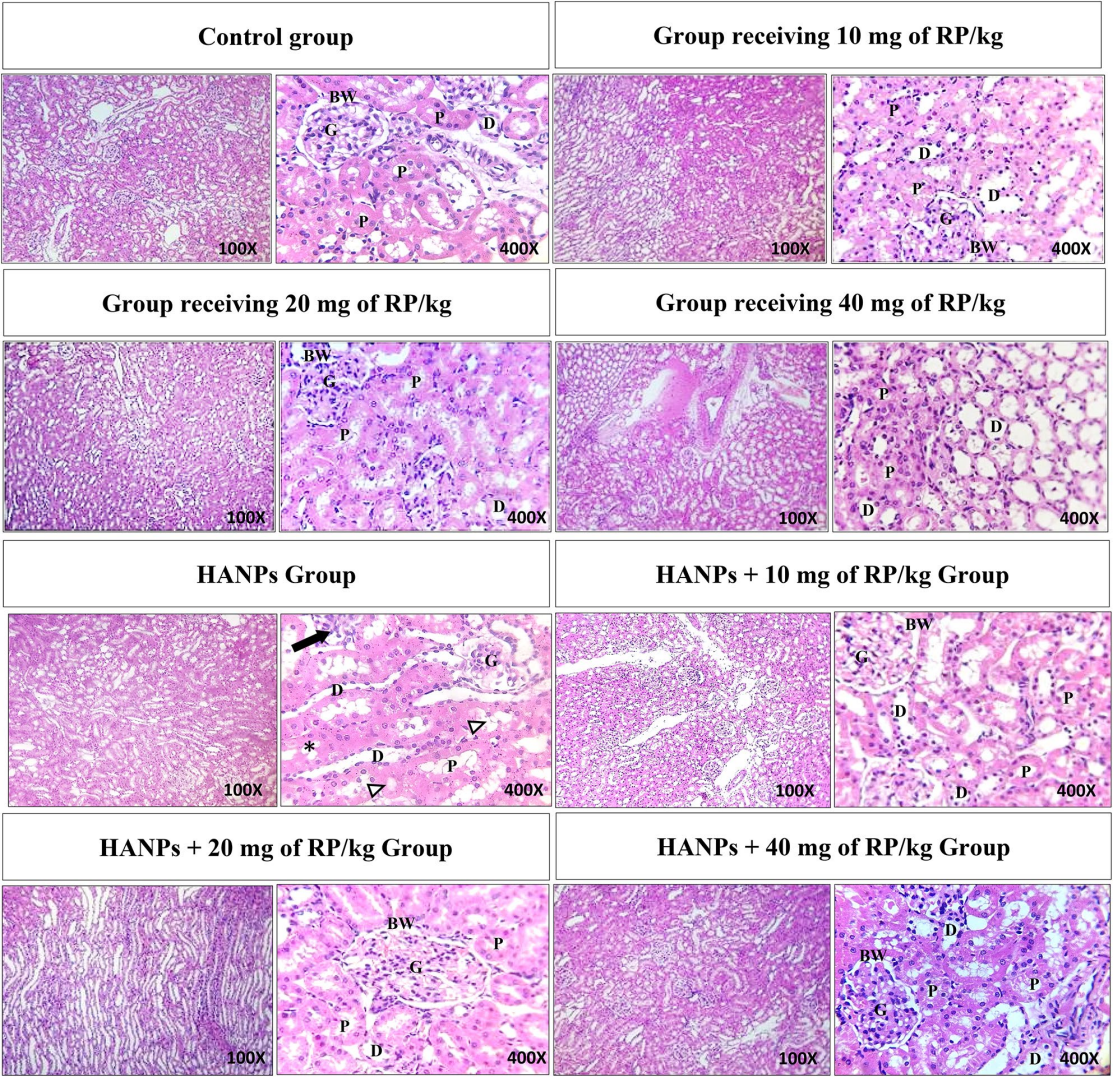
Representative photomicrographs of H&E stained kidney sections from the different experimental groups showing normal histoarchitecture in the control group as well as under the influence of different doses of RP as both the proximal (P) and the distal (D) convoluted tubules and the renal corpuscles including the Bowman’s capsules (BW) and the glomeruli (G) were normal in appearance. In HANPs-treated group, the black arrow indicates the leucocyte infiltration, note also the degenerative glomerulus, the Bowman’s space narrowing, the vacuolation (asterix) and the progressive degeneration of the renal tubules (arrowheads). Whereas kidney sections from rats receiving double treatment with HANPs and different doses of RP showed alleviated histological deterioration.

Examination of kidney samples showed normal histoarchitecture in control rats (Fig. 7). Regarding the kidney structure under the influence of different doses of RP, both the proximal and the distal convoluted tubules as well as the renal corpuscle enclosing a tuft of blood capillaries were normal in appearance (Fig. 7). In contrast, in HANPs-treated rats, renal corpuscles showed extensive congestion filling the glomerular capillary loops, some glomeruli were degenerated and others exhibited hypercellularity with leukocyte infiltration, edema exudate, and necrosis, and the Bowman’s space become narrow. The histological deterioration was alleviated in rats receiving double treatment with HANPs and different doses of RP except of minor ballooning degeneration in renal tubules. In addition, inflammatory cell infiltrations were visibly reduced in animals treated with both HANPs and RP.

## Discussion

Nanomedicine bridges the gap between life and materials sciences, after being sustained by different methods, terminologies and investigation subjects existing on each one of the separate research area^27^. In the present study, we focus on HANPs as one example of these materials having widespread applications in biomedicine. HANPs have shown high biocompatibility, high chemical stability, high mechanical strength in vivo^28^. Moreover, they have been used as vehicles for delivery due to their affinity to DNA, proteins, several drugs, and proper release activity^29^. However, these useful applications of HANPs do not exclude their adverse effects on various organs of the body. In the present study, we employed diverse tools to characterize both the HANPs as well as the naturally extracted RP from *Monascus purpureus.* Subsequently, an attempt was made to globally assess the effects of HANPs toxicity on the kidney of male rats, followed by investigating the impact of the co-administration of HANPs together with different doses of RP from *Monascus purpureus* on kidney functions and the overall physiological status of the animal.

The activity of HANPs is determined by their physicochemical properties (i.e., morphology, size, crystallinity) which are pivotal factors influencing HANPs toxicity. As complementary to TEM technique used, in the present study, to visualize the morphology and structure of particles at nano scale, spectroscopic techniques including FTIR and XRD were also applied. Obviously, FTIR spectroscopy qualitatively detect HANPs phases, although with much less precision than XRD. It has been shown that rod-like HANPs had advantages over spherical nanoparticles in cellular uptake^30^. Moreover, among the different types of HANPs morphology and crystallinity, Li et al.^31^ found that the rod-like one with a crystallinity of 45.60% was the most prominent to suppress the growth of cancer cells. This study confirms these previous findings^30–32^.

Under normal conditions, glomerular filtration by the kidneys removes the metabolic waste products including urea, creatinine, and uric acid from the blood. On the contrary, when the kidneys do not function properly or efficiently, these waste substances accumulate in the blood reflecting the drop in the renal health status. Herein, kidney dysfunction following HANPs administration were most likely manifested by an increase in the levels of urea and creatinine but not uric acid in the blood. A significant decrease of these levels in the plasma from rats receiving different doses of RP as compared to their corresponding in HANPs group suggests a potential protective activity. These findings agreed with our previous reports demonstrating the adverse effects of HANPS on the kidneys^33^. Moreover, the beneficial potential of the RP from *Monascus purpureus* have been previously revealed in vivo in rats fed with a high-fat diet^34^.

Meantime, renal toxicity was manifested by an imbalance of oxidant/antioxidant capacity in the tissues. In the present study, it has been shown that rats receiving HANPs exhibited an oxidative stress induction as revealed by the significant elevations of MDA and NOx levels as well as the increased activity of SOD and GSSG associated with a reduction in GSH level and GPx activity. Moreover, following HANPs administration, the GSH/GSSG ratio within tissues, which is often used as a marker of cellular toxicity^35^, displayed a drastic decrease, whereas the oxidative DNA marker, 8-OHdG, showed a significant increase.

These aberrations which were consistent with previous results depicting similar alterations in rats exposed to HANPs^36–38^, were greatly improved by the different doses of RP administered to rats.

In kidney progressive diseases, TNF-α and TGF-β1 are two pleiotropic cytokines that have been established as central mediators of inflammatory responses and renal fibrosis, respectively. On one hand, TNF-α can activate three diverse pathways including mitogen-activated protein kinase (MAPK), NF-κB, and cell death signaling pathways leading to severe glomerular and tubular renal damage^39^. On the other hand, kidney fibrosis represents the common pathway to end-stage renal failure^40^. Therefore, inflammation and fibrosis may complicate the pathologic processes and add deleterious effects to renal injury^41^. In the current study, HANPs administration induces an elevation of both TNF-α and TGF-β in rats associated with an increase in caspace-3, an apoptotic marker that could be induced either by renal damage or by upregulated TNF-α. The current results agree with the previously reported results^41,42^. However, different doses of RP administration would play a vital protective role against kidney damage in case of HANPs toxicity by diminishing the inflammatory, fibrotic, and apoptotic markers.

Toxicogenomics, preferentially the application of genomic data to elucidate or predict an organism’s response to a toxicant^43^, considered Kim-1 and lipocalin-2 as gene-based markers of kidney injuries^44,45^. Moreover, it has been reported that the increased expression of Kim-1 and lipocalin-2 could detect the damage before the onset of major histopathological changes in numerous animal models of renal injuries as well as in humans^45,46^. Interestingly, Kim-1 is a proximal tubule apical transmembrane protein having a phosphatidylserine receptor which enhances apoptotic bodies and necrotic debris phagocytosis. Elevated Kim-1 levels correlated with inflammation and fibrosis in the histological studies^47^. Our results have reported an induction in the kidney expression levels of these two molecular indicators of renal damage following HANPs administration. This finding is in accordance with the biochemical and histopathological alterations in kidney tissues of rats receiving HANPs.

In the current study, the observed HANPs-induced renal histopathological abnormalities including degenerated glomeruli with leukocyte infiltration, edema exudate, necrosis of both proximal and distal tubules, and decrease in urinary space and mild ballooning degeneration of renal tubules are consistent with previous results mentioning similar alterations in rats treated with HANPs^33^. Also, vacuolization and severe inflammatory reactions were distinguished. Conforming to previous investigations, we revealed that different doses of RP from *Monascus purpureus* could successfully alleviate the adverse effects of HANPs on kidney histoarchitecture^34^.

To summarize, this study brings insights for the research field of natural biopigment in the amelioration of the hazards of toxic elements. In our approach, we compared the effectiveness of different doses of RP from *Monascus purpureus* with potential protective benefits in mitigating the adverse effects of HANPs. Meanwhile, further research is still needed to help clarify the mechanism of action of RP and complement the knowledge about its beneficial characteristics.

## Data Availability

All data generated and analyzed in this study are included in this article.

## Abbreviations

8-OHdG: 8-Hydroxy-2-deoxyguanosine
DMSO: Dimethyl sulfoxide
EMCC: Egypt microbial culture collection
ELISA: Enzyme-linked immunosorbent assay
FTIR: Fourier transform infrared
GSH: Glutathione
GSSG: Glutathione disulfide
GPx: Glutathione peroxidase
HR-TEM: High resolution-transmission electron microscopy
HANPs: Hydroxyapatite nanoparticles
IACUC: Institutional animal care and use committee
ICLAS: International council for laboratory animal science
Kim-1: kidney injury molecule-1
LC-MS: Liquid chromatography-mass spectrometer
MDA: Malondialdehyde
NPs: Nanoparticles
NOx: Nitrite and nitrate
Kbr: Potassium bromide
qRT-PCR: Quantitative real time-polymerase chain reaction
ROS: Reactive oxidative species
RP: Red pigment
SOD: Superoxide dismutase
TGF-b: Transforming growth factor-beta
TNF-α: Tumor necrosis factor-alpha
UV-Vis: Ultraviolet-visible
XRD: X-ray diffraction
